# Applying Taguchi design and large-scale strategy for mycosynthesis of nano-silver from endophytic *Trichoderma harzianum* SYA.F4 and its application against phytopathogens

**DOI:** 10.1038/srep45297

**Published:** 2017-03-28

**Authors:** Shahira H. EL-Moslamy, Marwa F. Elkady, Ahmed H. Rezk, Yasser R. Abdel-Fattah

**Affiliations:** 1 Bioprocess development Department, Genetic Engineering and Biotechnology Research Institute, City of Scientific Research and Technology Applications, New Borg El-Arab City, Alexandria, Egypt; 2 Chemical and Petrochemical Engineering Department, Egypt-Japan University of Science and Technology, New Borg El-Arab City, Alexandria, Egypt; 3 Fabrication Technology Researches Department Advanced Technology and New Materials and Research Institute, City of Scientific Research and Technological Applications, New Borg El-Arab City, Alexandria, Egypt

## Abstract

Development of reliable and low-cost requirement for large-scale eco-friendly biogenic synthesis of metallic nanoparticles is an important step for industrial applications of bionanotechnology. In the present study, the mycosynthesis of spherical nano-Ag (12.7 ± 0.8 nm) from extracellular filtrate of local endophytic *T. harzianum* SYA.F4 strain which have interested mixed bioactive metabolites (alkaloids, flavonoids, tannins, phenols, nitrate reductase (320 nmol/hr/ml), carbohydrate (25 μg/μl) and total protein concentration (2.5 g/l) was reported. Industrial mycosynthesis of nano-Ag can be induced with different characters depending on the fungal cultivation and physical conditions. Taguchi design was applied to improve the physicochemical conditions for nano-Ag production, and the optimum conditions which increased its mass weight 3 times larger than a basal condition were as follows: AgNO_3_ (0.01 M), diluted reductant (10 v/v, pH 5) and incubated at 30 °C, 200 rpm for 24 hr. Kinetic conversion rates in submerged batch cultivation in 7 L stirred tank bioreactor on using semi-defined cultivation medium was as follows: the maximum biomass production (X_max_) and maximum nano-Ag mass weight (P_max_) calculated (60.5 g/l and 78.4 g/l respectively). The best nano-Ag concentration that formed large inhibition zones was 100 μg/ml which showed against *A.alternate* (43 mm) followed by *Helminthosporium sp.* (35 mm), *Botrytis sp.* (32 mm) and *P. arenaria* (28 mm).

Impact of nanoparticles on crop plants is a rising area of nanobiotechology research that needs to be cautiously explored. In recent years, engineered nanoparticles have achieved particular attention as a potential candidate for improving crop yield, resistance, and disease management technologies^1^. It is understood that the use of pesticides in agriculture is becoming more hazardous day by day. In order to replace such toxic materials by equally useful substances is an excellent choice, especially easily available metallic nanoparticles (MNPs) which are antimicrobial for most of the fungal and bacterial diseases in plants^2^. There are several methods reported for the synthesis of MNPs including physical, chemical and biological methods. Biological method is cheap, reliable, safe and non toxic over physical and chemical methods^1^,^3^. Among all microbial entities, the fungi were taking the centre stage of studies on biological generation of MNPs because of the tolerance and bioaccumulation^3^. Fungi are efficient secretor of extra cellular bioactive compounds & proteins and it can easily obtain its large scale production for MNPs^4^,^5^,^6^. Over the past decades, Ag NPs over an attractive considerable interest among the emerging nanomaterials^1^.This may be backed to the fact of their excellent and unique electromagnetic, optical, catalytic properties, and their antimicrobial effects against numerous microbes along with anti-proliferative effects compared with other metal nanoparticles^1^,^4^. Using microbes, especially their cell-free extracts, for the synthesis of nano-Ag can be advantageous compared with other biological processes because microbial resources are abundant in nature, are easy to culture, and have the potential to be scaled up for large-scale synthesis^7^,^8^,^9^.

Endophytic fungi are taxonomically and biologically diverse and dwell within robust plant tissue by having a symbiotic association. They have proven to be promising sources of new and biologically active natural products for exploitation in modern medicine, agriculture and industry^10^. Supra-molecular complexes of peptides to proteins, sugars to polysaccharides, terpenoids, polyphenols, glycosides, plant and microbial derived compounds, viral particles, etc., are being constantly explored for the biosynthesis of nano-Ag and novel carriers^1^,^11^,^12^,^13^,^14^. These proteins and biomolecules will often associate with nanoparticles that must be preventing the agglomeration and stabilize nanoparticles^15^. Fungi can produce nanoparticles both extracellularly as well as intracellularly however the exact mechanism is not understood completely. Putative mechanisms during intracellular synthesis include heavy metal binding to fungal cell wall by proteins or enzymes present on it via electrostatic interactions^16^. Furthermore, the metal ions are reduced by enzymes present in cell wall. This leads to aggregation of metal ions and formation of nanoparticles^1^. Extracellular synthesis assumed interaction of metal ions and release of enzyme mainly reductase with subsequent formation of nanoparticles in solution^17^. Extracellular synthesis of nanoparticles has advantages as it does not require lyses of fungal cell, downstream processing for recovery and purification of nanoparticles^15^,^18^,^19^. Whereas, in case of intracellular synthesis recovery and purification of nanoparticles from fungi biomass is tedious task and hence analytical equipments and long processing techniques are required^14^,^15^,^18^,^19^. Fungi of the genus *Trichoderma* are a very large microbial group that play a significant role in the environment and utilized in various industry branches mainly in the production of enzymes, antibiotics, and other metabolites^15^,^18^,^20^ reported that all of the *Trichoderma* species studied were efficient in production of nano-Ag specifically *T. virens*; so these species could also be used in future to explore applications of the nano-Ag^12^ have studied the growth inhibition effect of three types of nano-Ag against 18 different plant pathogenic fungi *in vitro*. Nano-Ag was used as an alternative to pesticides to control the *sclerotia*-forming phytopathogenic fungi^13^. The antifungal effect of doubly encapsulated nano-Ag solution against rose powdery mildew (leaf distortion, leaf curling, early defoliation, and reduced flowering) caused by *Sphaerotheca pannosa var rosae* was also studied^14^,^20^.

The green synthesis of nano-Ag involves three main criteria which must be evaluated based on the green chemistry perspectives include choice of solvent medium, variety of environmentally benign reducing agent and range of nontoxic substances for the Ag Nps stability^21^. The UV–Vis spectra recorded from the reaction medium at different pH values and the temperature also play an important role in acceleration the process of AgNPs production^22^. Different reports suggested that a variety of biomolecules are involved in biological synthesis of nanoparticle, such biomolecules are likely to be inactivated like polysaccharides and proteins under the extremely acidic conditions (pH 3.0) and start to work effectively in neutral and slightly alkaline conditions^17^. On another hand, the noticeable difference in the mixture colors obtained over the range of pH could be ascribed to a variation in the dissociation constants (pKa) of functional groups on the biomass that are involved^20^. Some reports also revealed that when the temperature is increased, the reactants are consumed rapidly leading to the formation of smaller nanoparticles^15^,^19^.

Taguchi is a simple and effective statistical method, which organizes a systematic experimentation to determine the near to optimum settings of design parameters for performance, quality, and cost. In this method, a large number of variables are studied with a small number of experiments using orthogonal arrays^17^. In the Taguchi approach, an orthogonal arrays and analysis of variance (ANOVA) are used for the analysis of experimentations. By using ANOVA, the effect of factors can be estimated and by orthogonal arrays the minimum number of experiments is needed. In this method variability of parameters is expressed by signal-to noise (S/N) ratio, which represents the ratio of desirable results (signal) to undesirable results (noise). In this statistical method the S/N ratio is used to measure the quality characteristic derivation from the desired value. The maximum S/N ratio is considered as the optimal condition as the variability is inversely proportional to the S/N ratio^23^. Taguchi method has been used for devising a suitable strategy to perform experiments as well as for quality control purposes in optimized conditions. Basically, the Taguchi experimental design is used to get information such as main effects of design parameters from minimum number of experiments. This method has been applied to nanoparticles for a limited number of syntheses, including silver^24^, zinc oxide^25^ and silica^26^, but this technique has not been applied to mycosynthesis of nano-Ag reaction.

The integration of engineering concepts with biological principles is one of the major contributions from “Biochemical Engineering” to the new biotechnology^27^. Engineering studies are always interested in consistently producing large quantities of natural bioactive products over long periods of time^28^,^29^. The best way to achieve this goal will be to grow the cells in a bioreactor where the cellular activity can be controlled efficiently. Historically, biochemical engineers have solved problems and accomplished designs necessary to implement at a large scale processes demonstrated in the laboratory involving enzymes, cells, and/or biological raw materials. This traditional scale-up role of biochemical engineers remains an important activity in practice and also in research^27^,^30^,^31^,^32^. The theoretical parts for microbial processing start with the concept of growth limiting nutrients. There are several mathematical relationships of specific growth rate coefficient to concentration of growth-limiting nutrient^33^,^34^. Medium and culture condition optimization has been reported in previous researches^35^, but quantitative analysis of nano-Ag production process and combining the analysis with optimization operation has been never reported. Kinetic modeling is considered to be a useful means for quantitative analysis, optimization and scaling-up of fermentation processes.

In this work; endophytic fungi were isolated from healthy organic tomato plant parts and screened for the mycosynthesis of nano-Ag by using exteracelular, periplasmic or cytoplasmic fractions. The *Trichoderma sp* isolate which appear relatively high frequencies of nano-Ag mycosynthesis will further choose for molecular identification. The obtained pure powder nano-Ag will characterized by XRD, TEM, SEM, EDX, FTIR and preliminary phytochemical analysis. The physical parameters need to be optimized for attaining maximum mycosynthesis of nano-Ag. Therefore the synthesis of nano-Ag via a simple, fast and eco- friendly method using the Taguchi design will evaluate to optimize essential nano-Ag mycosynthesis parameters. The behavior of fungal cell growth is a primary requirement in the mycosynthesis reaction will evaluate by using large scale batch submerged fermentation strategy. Finally antifungal activity of the optimized nano-Ago will be screened and determined against *Helminthosporium sp., Alternaria alternate, Phytophthora arenaria* and *Botrytis sp*.

## Methods

### Collection of plant material and isolation of fungal endophytes

Healthy organic tomato plants were collected from farm at city of scientific research and technological applications, Alexandria, Egypt **(2016);** average temperatures are between 30 to 36 °C. Tomato plants were cultivated in this locality with organic production system without the use of pesticides and mineral fertilizers. The collected samples were packed directly into sterilized polyethylene bags and transferred to the Bioprocess development lab, Genetic Engineering and Biotechnology Research Institute, City of Scientific Research and Technology Applications, New Borg El-Arab City, Alexandria, Egypt. Segments were taken from fresh healthy leaves, stems and roots. All segments were washed under running tap water to ensure for dust free and clean. Then, samples were washed thoroughly with sterile double distilled water (SDDW) and disinfected with 70% ethyl alcohol for one minute. Under aseptic conditions, samples were directly transferred to 2.5% of sodium hypochlorite solution for 4 min, followed by 70% ethyl alcohol for 1 min then with 70% alcohol followed by thorough rinsing with SDDW for four times. Samples were dried on sterile blotting paper and then cut into small segments. Sterile small segments were placed on rose Bengal agar medium with chloramphenicol^36^,^37^. All inoculated plates were then incubated at 26 ± 2 °C for a period of 10–20 days. The emergent mycelia were sub-cultured to PDA plates for purification. Pure fungal endophyte identification was performed according to morphological characteristics and microscopic examination which used to determine the reproductive and vegetative structures^36^,^38^,^39^,^40^.

### Screening of endophytic fungi extract fractions for mycosynthesis of nano-Ag

All the isolated endophytic fungi were screened for the mycosynthesis of nano-Ag. Isolated endophytic fungi were grown aerobically in malt medium with the following composition: 15 g/l malt extract, 15 g/l agar, and 0.2 g/l chloramphenicol in distilled water, pH 7.0 ± 0.4 and incubated in an orbital shaker at 26 ± 2 °C and agitated at 150 rpm for 7 days^41^. After that, these cultures were filtered using suction filtration system; so extracellular fraction collected and the cells used for prepare the periplasmic and cytoplasmic fractions. These fungal cells was washed extensively using Milli-Q deionized water to remove any medium component and 10.0 g (wet weight) of fungal cells were added to 100 ml distilled water in an Erlenmeyer flask and agitated again at 200 rpm for 48 hr at 30 °C. Then, the cell filtrate (periplasmic fraction) was obtained by filtering through Whatman filter paper^29^,^42^. The pellet was re-suspended in 5 ml cold 50 mM PBS at pH 7.0 and sonicated on ice with the power level set about 4–5, at 40–50% duty for 15–20 bursts. After centrifugation; the supernatant (cytoplasmic fraction) was taken and saved for mycosynthesis of nano-Ag. The filtrates were mixed with 100 m1 of 1 mM AgNO_3_ and incubated (30 °C) and agitated on orbital shaker at 150 rpm in the dark condition. Cell filtrate used as control under the same experimental conditions. After 24 hr of incubation, the formations of nano-Ag were screened by visual observation of color that changes to dark brown. Then it was further confirmed by subjecting the reaction mixture to UV– Visible spectrophotometer analysis. The spectrum was scanned by using a UV-Vis spectrophotometer (Spectronic 20, Arthur H. Thomas Co., USA) in the range of 200–800 nm.

### Molecular identification of the most potent endophytic *Trichoderma* isolate

Among the endophytic fungi isolates, one isolate appeared high frequency of nano-Ag mycosynthesis was chosen for further molecular identification. For determination the ribosomal internal transcribed spacer (ITS); the frozen fungal mycelia (1 g) were mechanically disrupted with liquid nitrogen in a mortar and pestle and mixed with 2.0 ml of 4.0 M guanidinium thiocyanate, 0.1 M sodium acetate pH 5.5, 10 mM EDTA, 0.1 M 2-mercaptoethanol. Extracts were clarified by centrifugation and supernatants were loaded into silica gel spin columns (Minipreps DNA Purification, Promega, USA). Columns were washed with 70% ethanol, 10 mM sodium acetate pH 5.5, and DNA eluted with 50 μl of 20 mM Tris-HCl, pH 8.5. ITS region was amplified by PCR using primer pairs ITS1 (5′-CTTGGTCATTTAGAGGAAGTAA-3′) and ITS4 (5′-TCCTCCGCTTATTGATATGC-3′). Amplification was performed in a 50 μl reaction mixture containing 100 ng of template DNA, 20 mM each dNTP, 1.5 mM MgCl_2_, 10 pmol of each primer, and 0.4 μl of 500 U Taq DNA polymerase. PCR amplification was carried out for ITS1 and ITS4 for 35 cycles of 94 °C for 1 min (denaturing), 55 °C for 1 min (annealing) and 72 °C for 150 s (extension). Initial denaturing temperature was 95ᵒC extended to 5 min and the final extension was for 10 min at 72 °C. PCR products were separated by electrophoresis in a 1% agarose gel run for 50 min in TAE buffer (40 mM Tris, 20 mM sodium acetate, 1 mM EDTA, pH 7.2) and viewed using a UV transilluminator after ethidium bromide staining. According to electrophoretic migration, the PCR products that corresponded to the ribosomal ITS which eluted from the gel using silica spin columns (DNA Clean & Concentrator, Zymo Research). The purified double strands PCR fragments were directly sequenced. The consensus sequences were employed to search for homologous sequences with the BLAST search program at the National Center for Biotechnology Information (NCBI; http://www.ncbi.nlm.nih.gov). Multiple sequence alignment was carried out using ClustalW2, and phylogenetic tree was constructed using the neighbor-joining (NJ) method.

### Screening of fungal bioactive metabolites

In these experiments, the comparison study performed between endophytic *T. harzianum* SYA.F4 and *T. harzianum* EMCC 540 strains for detecting the bioactive compounds which might be used in mycosynthesis of nano-Ag as an antifungal agent. This *T. harzianum* EMCC 540 strain provided from Egypt microbial culture collection (EMCC), microbiological resources center (Cairo MIRCEN).

#### Phytochemical analysis

Chemicals prospecting in ethyl acetate extracts of both fungal cell-free supernatant were performed to observe the presence of the following secondary metabolites: alkaloids, flavonoids, tannins, phenols, steroids, saponins, terpenoids and cardiac glycosides by using standard procedures as described by^8^,^43^,^44^,^45^,^46^,^47^,^48^,^49^.

#### Total Carbohydrate Assay

The total carbohydrate contents determined by using the total carbohydrate colorimetric assay kit (***Milpitas**, **CA*** **95035** ***USA***). Carbohydrate standard calibration curve was prepared with D-glucose with the range of 10–200 μg/ml. The amounts of carbohydrates were determined by using Varian Cary Bio 100 UV-Visible spectrophotometer at 490 nm against the blank.

#### Protein measurements

Proteins from the supernatants were quantified by using the commercial Bio-Rad Colorimetric Protein Assays kit which is based on the method of Bradford^50^ using bovine serum albumin as a standard. The assay was performed in 96-well microtitre plates in duplicates for each sample. The protein concentration was defined as the amount of extracellular proteins measured per L of fungal supernatant^51^,^52^,^53^. All analyses were done in triplicate in a temperature-controlled incubator.

#### Nitrate reductase assay

The enzyme-nitrate reductase in both culture filtrates with AgNO_3_ was assayed according to the procedure followed by^54^,^55^,^56^,^57^,^58^. An aliquot (5 ml) of 5-day fungal filtrate was mixed with 10 ml of assay medium (30 mM KNO_3_ and 5% iso-propanol in 0.1 M phosphate buffer of pH 7.5) and incubated at 25 °C for 1 hr in dark condition. After incubation, nitrites formed in the assay mixture were estimated by adding 1 ml of sulphanilamide and NEED (N-(1-naphthyl) ethylene diamine dihydrochloride) solutions in to it. The developed pink color was measured in an UV–vis spectrophotometer at 440 nm. The enzyme activity was finally expressed in terms of nM of nitrite/hr/ml.

### Characterization of mycosynthesized nano-Ag

The mycosynthesized nano-Ag was purified by the centrifugation of solution of nano-Ag at 14,000 rpm for 20 min with continuous washing the pellet with sterile Milli Q water. The final pellet was dried in hot air oven at 50 °C for 3 hr, and the pure powder obtained was used for XRD, TEM, SEM, EDX, FTIR analysis and other studies. X-ray diffraction patterns of the Ag nano-powders were obtained using Schimadzu 7000 diffractometer operating with Cu Kα_1_ radiation(λ = 0.15406 nm) generated at 30 kV and 30 mA with scan rate of 2°/min for 2θ values between 20° and 80° and its chemical composition was performed with an energy dispersiveX-Ray (EDS) analyzer combined with scanning electron microscope. The morphologies and size of nano-Ag were obtained from transmission electron microscope (TEM): JEOL JEM2100F- Japan and scanning electronmicroscopy (SEM) (JEOL JSM 6360LA, Japan). Finally FTIR analysis was carried out to study the functional groups possibly involved in the synthesis and stabilization of nano-Ag. The FTIR spectrums were measured using Shimadzu FTIR-8400 S, Japan, over the wave length range 400–4000 cm^−1^.

### Taguchi Design for optimization of nano-Ag stable mycofabrication production

The Taguchi method is used for the experiment and a generic signal-to-noise (S/N) ratio is used to quantify the current variation. This method seeks to improve product or process quality by reducing the mean squared deviation. Depending on the particular type of characteristics involved, different S/N ratios may be applicable, including “the lower the better” (LB), or “the higher the better” (HB). The S/N ratios are calculated using the equations no. 1^59^,^60^:

### The higher the better (maximize)


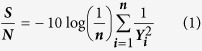


where **n** is the number of observations and **y** is the observed data; the **S/N** ratio is expressed using a decibel scale (**dB**). An analysis of variance (ANOVA) is performed to determine which process parameters are statistically significant. The S/N ratio and ANOVA analyses allow the prediction of the optimal combination of process parameters^60^. A confirmation experiment is then conducted to verify the optimal process parameters determined from the parameter design. An ANOVA and an *F*-test are used to analyze the experimental data by using Eq. 2, 3 and 4,^59^,^60^,^61^.


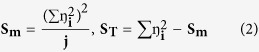



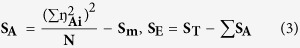



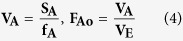


where **S**_**T**_ is the sum of squares due to the total variation, **S**_**m**_ is the sum of squares due to the means, **S**_**A**_ is the sum of squares due to parameter **A** (precursor conc. (AgNO_3_), reductant conc. (supernatant) and agitation speed), **S**_**E**_ is the sum of squares due to error, **ŋ**_**i**_ is the **ŋ** value of each experiment (**i** = 1,….,16), **J** is the number of experiments in the orthogonal array (in this work, J = 16), **ŋ**_**Ai**_ is the sum of the *i*^th^ level of parameter A (i = 1, 2, 3, 4), **N** is the repeating number of each level of parameter **A**, **f**_**A**_ is the degree of freedom of parameter **A** and **V**_**A**_ is the variance of parameter A. The predicted *S/N* ratio with the optimal level of the design parameters can be calculated by using Eq. 5.


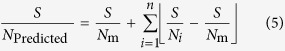


where ***S*****/*****N***_**m**_ is the total mean ***S*****/*****N*** ratio and ***S*****/*****N***_**i**_ is the mean ***S*****/*****N***ratio at the optimal level; ***n*** is the number of the main design parameters that affect the quality characteristic^59^. In this study, the synthesis of nano-Ag particles via a simple, fast and eco- friendly method using the Taguchi design was evaluated. Orthogonal array of L16 type was applied as an experimental design to analyze the results and to determine optimum conditions for mycosynthesis of nano-Ag. To optimize these nano-Ag mycosynthesis parameters, a Taguchi design approach were applied by using the following steps: selection of orthogonal array, assignment of factors, experimental setup, and statistical analysis for the final data. ANOVA analysis was used to determine the significant parameters in the process (main effect plot, S/N ratio and analyze the optimal level for all tested variables), and finally Interpretation and experimental conclusion. The reaction condition of the tested nano-Ag mycosynthesis parameters were ranged as the following: precursor conc. (0.01, 0.5, 1.0 and 1.5 M), diluted reductant (10, 50, 80 and 100 v/v) and agitation speed (50, 100, 150 and 200 rpm). The experiments were performed in triplicate and all flasks were kept for 24 hr at room temperature (28 ± 2 °C). After conducting experiments, the optimum conditions of reaction parameters were selected by measuring the nano-Ag dry mass weight as the final response. For statistical calculations and modeling Minitab (version 16) software was used. The Taguchi method uses a statistical measure of performance called signal-to-noise (*S/N*) ratio which was used in this work to evaluate the quantity of the myco-synthesized nano-Ag^17^,^21^. After performing the statistical analysis of the *S/N* ratio, an analysis of variance (ANOVA) needs to be employed in order to estimate errors and to determine the relative importance of various parameters.

### Scale-up production of nano-Ag biosynthesized mass weight

In the present study, several naturally available substrates of liquid media were tested for biomass production of *T. harzianum* which should be amenable to easy & cheap mass multiplication^62^. The success of nano-Ag biosynthesized as biocontrol agent depends not only the isolation, characterization and pathogenicity but also on the successful mass production of the fungal metabolites in lab^2^,^63^. Large scale availability of the fungal biomass is a primary requirement in the biosynthesis of nano-Ag programmed^5^,^28^,^30^,^35^,^62^,^64^.

#### Media evaluation

The composition of the pre-culture medium was adapted from the previous publications^4^,^7^,^27^,^28^,^30^,^35^,^65^,^66^. The fermentation was carried out in 250 ml shake flasks using a six different medium. **Medium (1)** consisted of (g/l): 9.0 Oat (Sigma), 5.0 yeast extract, 1.0 NaNO_3_, 1.0 KH_2_PO_4_, 1.0 peptone, 0.3 MgSO_4_·7H_2_O, pH 5.5, 4.2% (w/v) (NH_4_)_2_SO_4_ and 2% (w/v) glucose which used as sole carbon source. **Medium (2)** consisted of (g/l) 20.0 cellulose, 5.0 sucrose, 2.0 soybean meal, 5.0 wheat bran, 1.0 Tween 80, 100 ml of a mineral solution that consisting of (g/l): KH_2_PO_4_ (5.0), (NH_4_)_2_SO_4_ (14.0), MgSO_4_7H_2_O (6.0),CO (NH_2_)_2_ (6.0), CaCl_2_ (6.0), FeSO_4_7H_2_O (0.1), MnSO_4_H_2_O (0.03), ZnSO_4_7H_2_O (0.03), and CoCl_2_6H_2_O (0.04). **Medium (3)** consisted of (g/l) 1.0 ml Tween80; 0.3 urea; 2.0 KH_2_PO_4_; 1.4 (NH_4_)_2_SO_4_; 0.4 CaCl_2_·2H_2_O; 0.3 MgSO_4_·7H_2_O; 1.0 proteose peptone; 5.0 mg/l FeSO_4_·7H_2_O; 1.6 mg/l MnSO_4_·4H_2_O; 1.4 mg/l ZnSO_4_·7H_2_O; 2.0 mg/l CoCl_2_·6H_2_O; and 3% (w/v) glucose as the sole carbon source. **Medium (4)** consisted of (g/l) 20.0 cellulose, 5.0 KH_2_PO_4_, 5.0 (NH_4_)_2_SO_4_; 1.0 MgSO_4_.7H_2_O; 1.0 NaCl; 5.0 mg/l FeSO_4_.7H_2_O; 1.6 mg/l MnSO_4_; 3.45 mg/l ZnSO_4_.7H_2_O and 2.0 mg/l CoCl_2_.6H_2_O. **Medium (5)** consisted of (g/l) 50.0 microcrystalline cellulose, 17.0 corn steep liquor, 5.0 (NH_4_)_2_SO_4_, 6.0 KH_2_PO_4_, 1.0 MgSO_4_, 2.5 CaCO_3_, 2.5 glycerol, 2.0 ml Tween80, and initial pH 5.0. **Medium (6)** consisted of (g/l) 2.0 diammonium tartrate, 4.0 MgSO_4_.7H_2_O, 14.0 K_2_HPO_4_, 0.2 CaCl_2_, 4.0 NaHPO_4_, 3.0 yeast extract, 2.0 ml trace elements and 6.0glucose. The flasks containing 100 ml fermentation medium were sterilized at 121 °C for 15 min and inoculated by 5discs of vegetative cells. The temperature was controlled at 26 ± 2 °C for 4 days with shaking in orbital rotary shaker (200 rpm). The biomass was harvested through fungal growth by centrifuge, followed by substantial washing with distilled water in order to remove any medium component from the biomass. These cells were prepared for determination of mass weight/incubation time.

#### Cultivation in shake-flask

From the previous step the selected medium were used for large scale production. A conidia suspension prepared by adding 20 ml of sterilized distilled water and Tween80 to the grown PDA plates, which transferred to Erlenmeyer flasks containing 50 ml of pre-culture medium and incubated for 96 hr at 30 °C on a rotary shaker at 200 rpm^30^,^35^,^67^. A volume of 20 ml of this pre-culture was transferred to 1000 ml Erlenmeyer flasks containing 500 ml of the production medium. The biomass was harvested, followed by substantial washing with distilled water, dried in oven at 70 °C and the final fungal mass weight was determined. And the extracellular fractions used for mycosynthesis of nano-Ag by using the final optimized conditions; 0.01 M AgNO_3_ and diluted supernatant (10 v/v pH 5) at 30 °C and 200 rpm for 24 hr. Nano-Ag dry mass weight prepared as the following steps; formed nano-Ag particles was harvested by centrifugation at10000 rpm for 20 min, washed three times with distilled water and dried in oven at 50 °C for 24 hr.

#### Cultivation in stirred tank bioreactor

A 7.0 L BioFlo 310 Fermenter (New Brunswick Scientific Co., USA), equipped with automatic control of temperature, pH, agitation rate and aeration rate, inoculated with 10% (v/v) inoculums from the fungal pre-culture. The aeration rate was adjusted to not let the dissolved O_2_ level in the culture medium drop below 20% of air saturation. The pH (5.5–6.0) was controlled at pre-set values using either 4 M HCl or aqueous 4 M NaOH solution. Foaming was controlled by using polypropylene glycol antifoaming agent at initial concentration of 5.0 ml/l^6^,^35^,^65^. Samples were periodically removed, centrifuged at 15,000 g for 25 min (at 4 °C) for measurements of fungal mass weight and bio-fabricated nano-Ag dry mass weight as described in “the cultivation in shake-flask” section.

#### Kinetic theory for bioprocessing of fungal cells

The behavior of fungal cell growth can be described kinetically by using batch cultivation mode^32^. Therefore, the growth kinetic relationship is affected by many parameters such as biomass yield coefficient (***Y***_***X/S***_), Maximum biomass (**X**_**max**_), doubling time (**t**_**d**_), Maximum nano-Ag production (**P**_**max**_), nano-Ag production yield coefficient (**Y**_**P**_) and maximum specific growth rate **(μ**_**max**_).

#### Modeling of batch fermentation

The most common culture system is the batch culture, due to its simplicity and low cost. This is a closed system in which there is no input or output of materials^6^,^32^. The microbial population cell density increases constantly until exhaustion of some limiting factor, while other nutrient components of the culture medium decrease over time^65^. Any products produced by the cells during growth also increase in concentration in the culture medium. In batch culture, it was important to calculate growth yield for fungal cells during cultivation on carbon source. Yield coefficient was calculated based on the amount of glucose consumed^68^. The equation no. 6 was used to calculate growth yield:


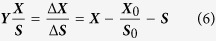


where: ***Y***_***X/S***_ Biomass yield on substrate, **X** Cell concentration, **X**_**0**_ Initial cell concentrations, **S** Substrate, **S**_**0**_ Initial substrate concentration. **X** and **X**_**0**_ are biomass concentrations (g/l) at measuring time **t** and initial time **t**_**0**_ respectively. **S** and **S**_**0**_ are the consumed amounts of carbon source (g/l) at the same times mentioned previously. In logarithmic growth phase, cell biomass density increases exponentially with time **t** and specific growth rate **μ (h**^**−1**^) is independent of nutrient concentration. According to ref. 68, the exponential growth is characterized by a straight line on a semi logarithm plot of ln *X* versus time. The equation no. 7 and 8 calculated by integration of equation 6 which yields


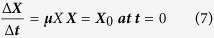



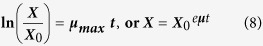


The equations no. 9 and 10 was used to calculate doubling time of cells:


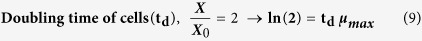



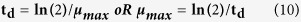


Finally the most common kinetic model for cell growth was used to determine the maximum specific cell growth rate (Eq. 11).


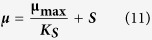


where; **μ** specific cell growth rate (hr^−1^), **μ**_**max**_ maximum specific cell growth rate (hr^−1^), **S** substrate concentration (g/l), **K**_**S**_ Saturation constant (g/l) = **S** when **μ = **1/2 **μ**_**max**_.

### Application of nano-Agas antifungal agent against some phytopathogenic fungi *in vitro*

Antifungal activity was screened against *Helminthosporium sp., Alternaria alternate, Phytophthora arenaria* and *Botrytis sp*., by agar well diffusion method. PDA plates were prepared and swabbed using sterile L-shaped glass rod with 100 μl of mature spore and/or conidial suspension of individual pathogenic fungal strains. The wells were made by using sterile cork borer (5 mm) wells was created into the each Petri-plates. Different concentration of nano-Ag (50, 100, 150, and 200 μg/ml) were used to assess the maximum inhibition zone. Then the plates were incubated at 30 °C for 96 hr, the inhibition zone measured in millimeter (mm). Analysis of variance (ANOVA) was used to determine whether there was a significant difference between replicates. ANOVA demonstrated either a significant difference (*p* < 0.05) or no significant difference (*p* > 0.05) for the results obtained. Where a significant difference was observed, the least significant difference (LSD) test was used to determine where the differences occurred.

## Results

### Screening of endophytic fungi for mycosynthesis of nano-Ag and molecular identification

**Figure 1:**
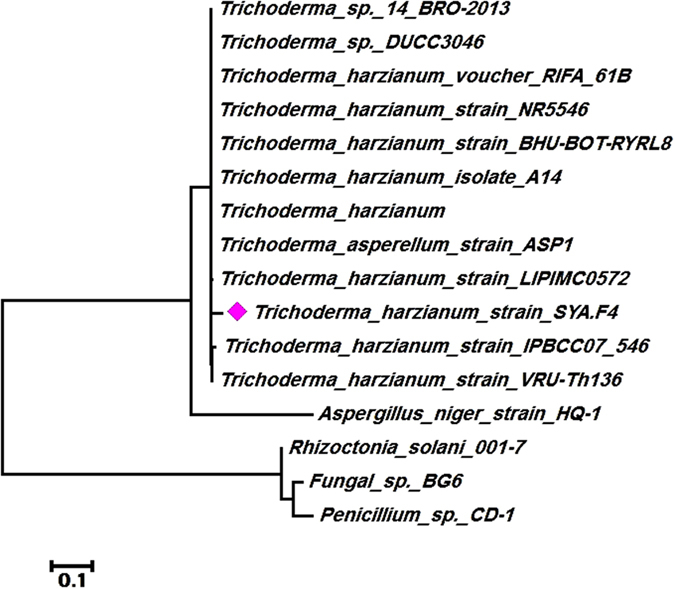
Neighbour-joining (NJ) analyses of phylogenetic relationship between the *T. harzianum* strain SYA.F4 (KX084391) and ITS-rDNA of related fungal strains retrieved from NCBI GenBank. Sequence divergence is indicated by the scale bar. Samples giving identical ITS1-5.8S-ITS2 gene sequences were assigned to an arbitrary group and are represented in the tree by their group and species designation. Marked strain is our GenBank submitted strain.

The formation of nano-Ag was preliminarily confirmed by visual observation of color change to yellowish brown and then dark brown. Mycosynthesis of nano-Ag were examined by UV-visible-spectroscopy, to monitor and assess its production, as one of the most widely used techniques for structural characterization of nanoparticles. A total seven endophytic isolates out of thirty isolates showed a reasonable color change of the cell filtrate. Nano-Ag producing taxa were *Trichoderma viz., T. asperellum, T. harzianum, T. longibrachiatum, T. pseudokoningii, T. virens,* and *Trichoderma sp,* but the nano-Ag spectra that showed from *Trichoderma* isolate F4 was the highest absorbance. In addition to the morphological characterization, molecular identification was carried out to confirm the identification of most promising endophytic fungal isolate. The endophytic *Trichoderma* F4 was characterized by the PCR amplification of 18 S rRNA gene using ITS primers. The amplified PCR product was around the size of 600 bps. The Sanger’s dideoxy nucleotide sequencing of amplified ITS region (ITS 1–5.8S-ITS 2) of 18 S rRNA gene resulted in 639 bp nucleotide sequence. The Blastn analyses, pairwise and multiple sequence alignment revealed 98–100% identity with the sequences of *Trichoderma harzianum* strains and is designated as *Trichoderma harzianum* strain SYA.F4 and has been deposited in NCBI GenBank (Accession Number KX084391, https://www.ncbi.nlm.nih.gov/nuccore/1036392272). Multiple sequence alignment was carried out using ClustalW2 with default parameters. Phylogenetic tree was constructed by the neighbour-joining (NJ) method with nucleotide pairwise genetic distance Fig. 1.

### Efficiency of secondary metabolites for nano-Ag mycosynthesis

In these experiments, secondary metabolites produced from endophytic *T. harzianum SYA.F4* compared with others from *T. harzianum* EMCC 540 strain (Cairo MIRCEN) to assess its nano-Ag mycosynthesis efficiency.

#### Localization of the most potent bioactive metabolites

**Figure 2:**
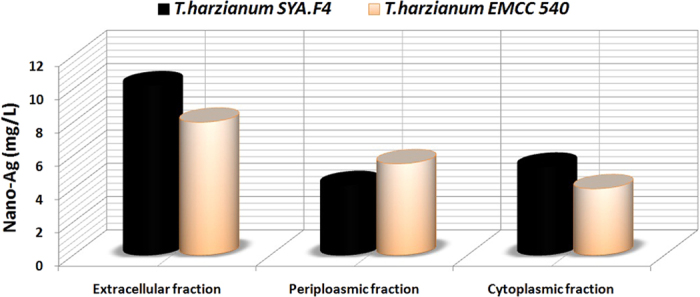
Dry weight of nano-Ag powder mycosynthesized by using the extracellular, periplasmic, and cytoplasmic fractions from *T. harzianum* strain SYA.F4 and *T. harzianum* EMCC 540.

The cellular localization of the bioactive metabolites which used for nano-Ag mycosynthesis from two cultures was determined by measuring the nano-Ag mass weight at different localities; to facilitate verification, a small-scale analysis of total cell protein and active compounds which founded in the extracellular, periplasmic and cytoplasmic fractions. All these fractions produced nano-Ag from both fungal culture but the highest dry mass weight produced from extracellular fraction which extracted from *T. harzianum* SYA.F4 (10.5 mg/l) compared with *T. harzianum* EMCC 540 strain (8 mg/l) as shown in Fig. 2.

#### Ultra violet-visible (UV-Vis) spectra analysis

**Figure 3:**
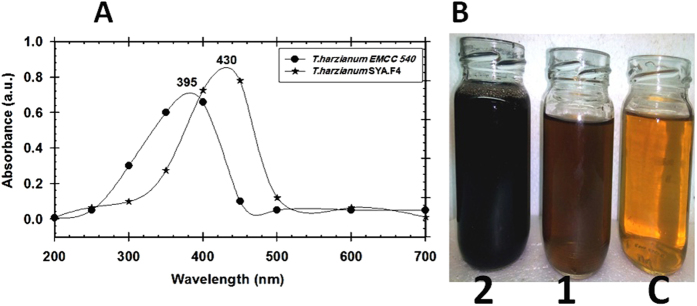
(**A**) UV–Vis analysis of the nano-Ag mycosynthesized from *T. harzianum* strain SYA.F4 and *T. harzianum* EMCC 540, (**B**) Mycosynthesis of nano-Ag after 24 hr and; (**C**) Crude cell filtrate without AgNO_3_ (**1**) Nano-Ag produced from *T. harzianum* EMCC 540,(**2**) Nano-Ag produced from *Trichoderma harzianum* strain SYA.F4.

In Fig. 3 a strong UV-Vis absorption peak recorded from the studied endophytic *T. harzianum* SYA.F4 and *T. harzianum* EMCC 540 strain after 24 hr of incubation centered at 430 and 395 nm which indicates the formation of nano-Ag and its intensity indicated the amount of produced sliver nanoparticles (brown color).

#### Phytochemical analysis of the bioactive metabolites

The results of qualitative analysis of the phytochemicals were summarized in Table 1. Preliminary phytochemical analysis revealed the presence of Alkaloids, Flavonoids, Tannins, Phenols, and Cardiac glycosides in *T. harzianum* SYA.F4 extract but Alkaloids and Phenols indicated in *T. harzianum* EMCC 540 strain extract. Terpenoids observed in *T. harzianum* EMCC 540 strain extract but absent in endophytic *T. harzianum* SYA.F4 and finally we note the absence of Steroids and Saponins in both extracts.

#### Nitrate reductase, Total protein and Carbohydrate concentrations

The results of quantitative measurements of these metabolites were summarized in Table 2. In this study highly nitrate reductase and protein concentration were detected and calculated in *T. harzianum SYA.F4* (320 nmol/hr/ml and 2.5 g/l respectively) but the highly carbohydrate concentration was recorded in *T. harzianum* EMCC 540 strain (75 μg/μl).

#### Screening for more effective nano-Ag as antifungal agent

**Figure 4:**
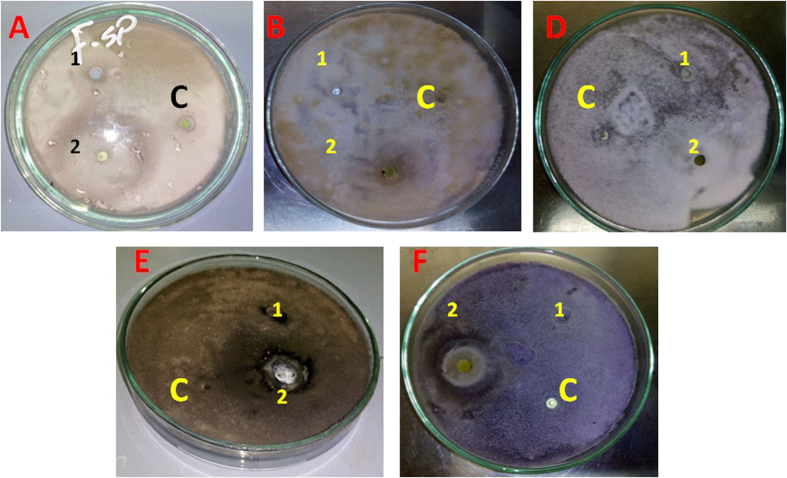
The Antifungal activity of nano-Ag (100 μg/ml) against the growth of phytopathogenic fungi *in vitro*. (**A**) *Fusarium sp.* (**B**) *Rhizoctonia solani* (**D**) *Fusarium proliferatum;* (**E**) *Botrytis cinerea;* (**F**) *Fusarium oxysporum* (**C**) Crude cell filtrate without AgNO_3_ (**1**) Nano-Ag produced from *T. harzianum* EMCC 540, and (**2**) Nano-Ag produced from *Trichoderma harzianum* strain SYA.F4.

Antifungal activity of nano-Ag myco-synthesized from both strains was screened against some phytopathogenic fungi (*Fusarium proliferatum, Fusarium sp., Botrytis cinerea, Rhizoctonia solani* and *Fusarium oxysporum)* by agar well diffusion method. In this experiment antifungal activities of nano-Ag mycosynthesized from both strains observed; but the highly antifungal activities were detected by using nano-Ag produced from *T. harzianum SYA.F4* extract against *Fusarium sp*. followed *Botrytis cinerea, Fusarium oxysporum* and *Rhizoctonia solani*, but no response appeared against *Fusarium proliferatum* by using both nano-Ag as shown in Fig. 4.

### Characterization of mycosynthesized nano-Ag

Nanoparticles have optical properties that are responsive to size, shape, concentration, agglomeration state, and refractive index near the nanoparticle surface, which studied by using an important analysis (UV-Vis spectroscopy, SEM, TEM, EDX, XRD and FTIR) for identifying, characterizing, and studying these materials.

#### Scanning and transmission electron microscopy (SEM & TEM)

**Figure 5:**
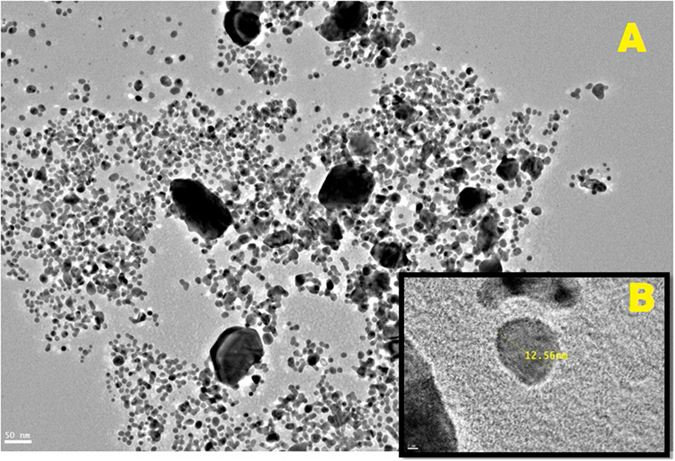
Mycosynthesized nano-Ag from *T. harzianum* strain SYA.F4 characterized by (**A**) SEM that shown nano-Ag spherical shape and (**B**) TEM shown its average size is 12.7 ± 0.8 nm.

These analyses were performed to determine the size and shape of the mycosynthesized nano-Ag. The obtained micrographs show that nanoparticles were roughly spherical in shape, well dispersed and its size was 12.7 ± 0.8 nm Fig. 5.

#### Energy dispersive X-ray analysis (EDX)

**Figure 6:**
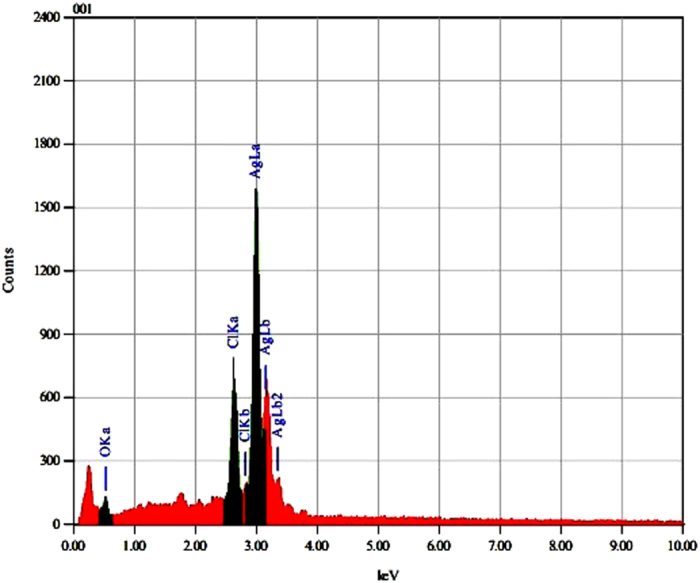
Energy dispersive X-ray analysis of the mycosynthesized nano-Ag from *T. harzianum* strain SYA.F4 shows nano-Ag peak at approximately 3 keV.

It is a compositional analysis technique. This analysis gives qualitative as well as quantitative status of elements that may be involved in formation of nanoparticles. The elemental profile of nano-Ag mycosynthesized from *T. harzianum* strain SYA.F4 showed typical characteristic elemental peak at approximately 3 keV, which was attributed to the SPR of the Ag nano-crystals and confirms the formation of silver nanoparticles Fig. 6.

#### X-ray diffraction (XRD) analysis

**Figure 7:**
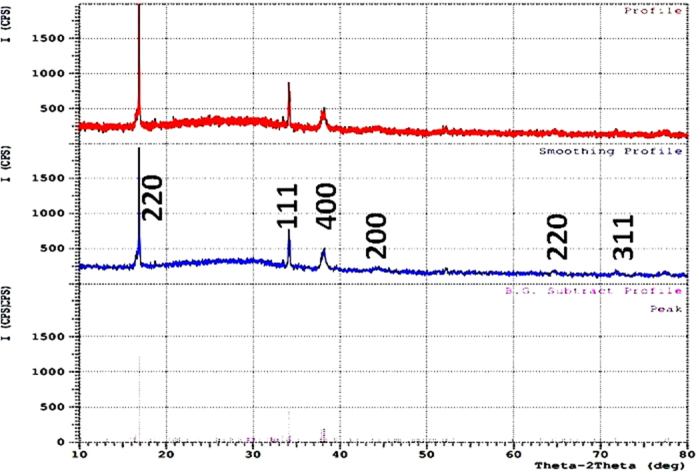
X-ray diffraction patterns of the mycosynthesized nanoAg from *T. harzianum* strain SYA.F4.

It is the main method for crystallographic characterization for bulk, nano and thin film materials. The XRD pattern of the mycosynthesized nano-Ag was shown in Fig. 7. XRD pattern revealed six diffraction peaks at 19.2, 34.31, 39.58, 43.71, 65.71, and 72.92 could be indexed to (220), (111), (400), (200), (220), and (311) planes, respectively. All these peaks corresponding to face-centered cubic (FCC) structure of metallic silver (JCPDS file no. 00–004–0783). Thus, XRD pattern obtained for the nano-Ag that revealed its crystalline nature and consisted with many earlier reports of the nano-Ag synthesized by fungal extracts.

#### Identifications of functional group using FT-IR

**Figure 8:**
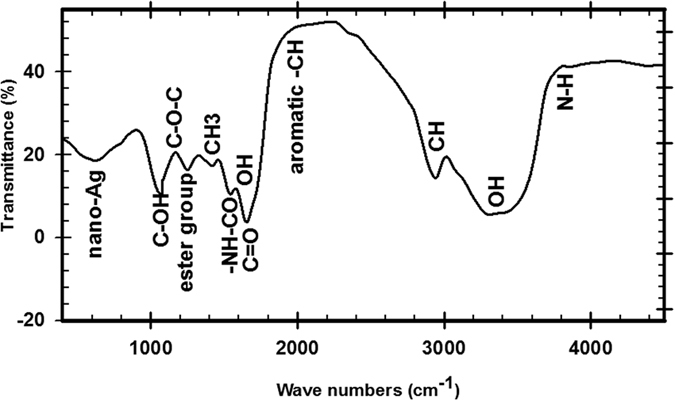
FTIR analysis of of nanoAg obtained from *T. harzianum* strain SYA.F4.

FTIR measurement of the freeze dried nano-Ag powder was carried out to identify the possible interactions between silver and bioactive molecules, which may be responsible for synthesis and stabilization (capping material) of nano-Ag by the fungal cell filtrate Fig. 8. As shown in FTIR analysis the intensive peaks of the sample at 3843.61, 3597.71, 3432.2, 2925.3, 2865.3, 2080.5, 1735.3, 1631.61, 1654, 1452.6, 1359.3, 1325.6, 1252.3, 1115, 1025 and 567.2 cm^−1^ which corresponding to -NH group of amines, -OH group of phenols, N–H stretching of the secondary amide of the protein, C–H stretching of methylene groups of the protein, aromatic-CH stretching, the stretching vibration of C = O, -NHCO of amide, the stretching vibrations of C = C or the O–H bending mode, the symmetrical and asymmetrical bending vibrations of –CH3, C–N stretching vibrations of aromatic amines, the ester group, the stretching variation of C–O–C, C–OH of the phenols or carbohydrates and nano-Ag respectively.

### Taguchi design for optimization of nano-Ag mycosynthesis reaction

**Figure 9:**
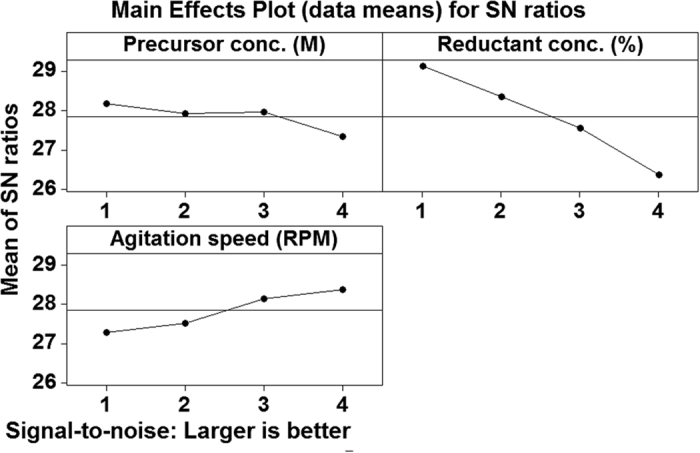
Response graph of S/N ratio for larger-the-better analysis for nano-Ag production. The horizontal axis shows the different levels of each significant factor. The lines represent the trend of each factor with respect to different levels.

In this study, the production of nano-Ag is affected by various parameters such as the reductant concentration, precursor concentration and agitation speed used during mycosynthesis reaction. The interaction between these variables is complex but by using Taguchi design method makes it possible to develop an acceptable formulation using minimum raw materials and save time. In this experiment; the Taguchi orthogonal arrays (OA) method was used to identify the optimal conditions and to select the reaction parameters that have the most significant effect on the dry mass weight of nano-Ag. Taguchi OA was applied by three parameters and four levels of each parameter Table 3. The orthogonal arrays of the L16 **(4**^**3**^) type were used, indicating that 16 experiments were required to study three the parameters at four levels involved with the target output parameter being the final dry weight of nano-Ag and the average S/N ratio of each factor at each level as shown in Table 4. The main effect of different parameters on the final dry weight of nano-Ag at four different levels (1, 2, 3 and 4) was investigated Fig. 9. The multiple linear regression model of Taguchi design screening method describes the relationship between the nano-Ag production from *T. harzianum* strain SYA.F4 and 3 independent variables. According to the main effect analysis, the three variables were analyzed using Taguchi design linear multiple regression analysis method and the % confidence level were calculated from the formula:


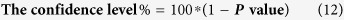


**Figure 10:**
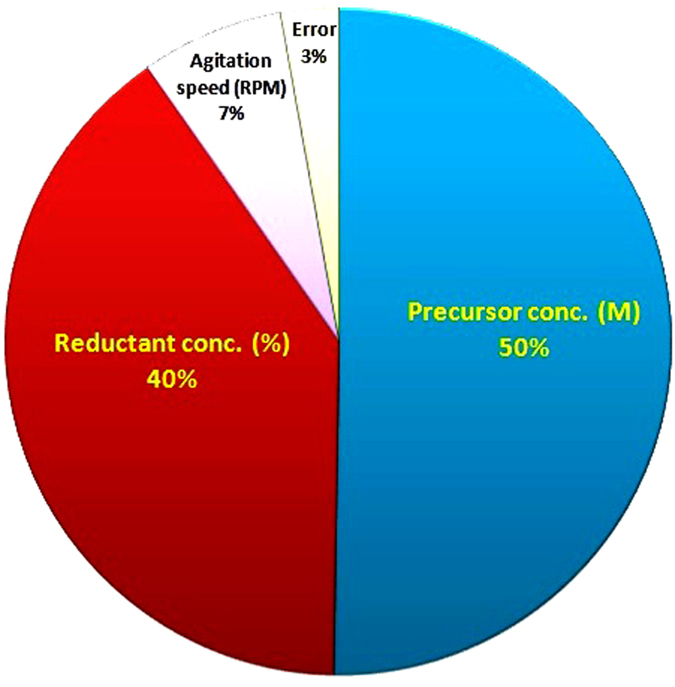
Percent distribution of each factor to the response for nano-Ag production.

**Figure 11:**
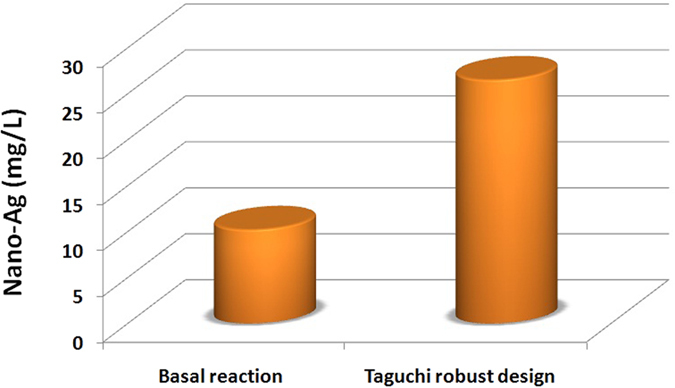
Comparison study between the basal reaction and optimized reaction indicated by final dry weight of nano-Ag (mg/l) which myco-synthesized by *T. harzianum* strain SYA.F4 extract.

Table 5 shown the *R-*squared and adjusted *R-*squared statistic which indicates that the model as fitted explains 99% of the variability in the nano-Ag mycosynthesis parameters. The *P-* value from the ANOVA analysis for each response was determined to analyze the relationship between the variables at 95% or higher confidence level as summarized in Table 6. The analysis of variance using ANOVA test was generated and summarized in Table 7. This indicates that there is a statistically significant relationship between the variables at 99.00% confidence level. In the Taguchi method, the signal/noise (S/N) ratio is a measure of signal quality and deviation from the desired value. The term “signal” represents the desired value (mean), whereas “noise” represents an undesired value (standard deviation from mean) for output characteristics. Taguchi method was used to identify the optimal reaction conditions and influencing parameters on dry weight of nano-Ag and for this purpose the obtained experimental data were processed with the “larger the better” quality characteristics. In this experimental setup, we used higher S/N ratio is better. The S/N ratio was determined and found that the interpretation of plot of S/N ratios is opposite to main effect plot. The results of S/N ratio study showed that at higher S/N ratio larger dry mass weight of nano-Ag was obtained and found that the precursor conc. (M) and reductant conc (v/v) are a significant parameter for the control of dry weight of nano-Ag while agitation speed had insignificant effects on these results. The level average graph of the raw data is illustrated in Fig. 10 which shown the contribution percentage of all factors for nano-Ag production. Based on the S/N and ANOVA analyses, the optimal parameters for nano-Ag production are the precursor conc., at 0.01 M, the diluted reductant at 10 (v/v) and the agitation speed at 200 rpm. The final step in the Taguchi method is to predict and verify the improvement of the quality characteristic using the optimal level of the design parameters. Finally, the estimated dry mass weight of nano-Ag can be obtained as 28.8 mg/l. The predicted *S/N* ratio for standard deviation can also be calculated by the same procedure. The predicted value with the experimental results using the optimal conditions was 29.6 mg/l, so very good agreement between the predicted and experimental nano-Ag production is observed. Consequently, nanoparticles production and its standard deviation can be increased (3 times larger than basal condition) and improved through the Taguchi method approach as shown in Fig. 11.

### Scale-up production of nano-Ag mass weight mycosynthesized from *Trichoderma harzianum* strain SYA.F4 extract

#### Media evaluation

**Figure 12:**
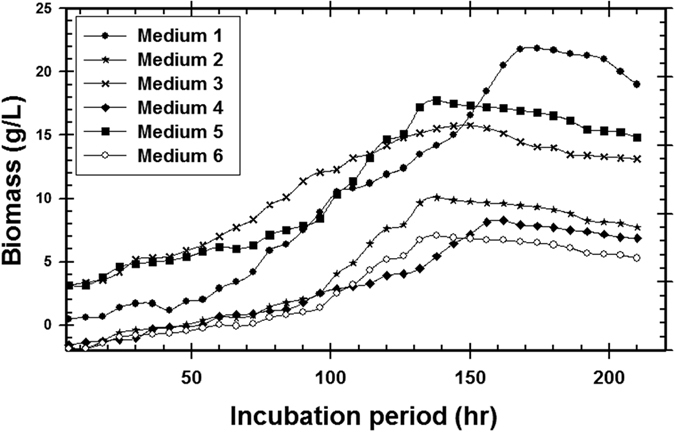
The time course of biomass production of *T. harzianum* strain SYA.F4 in various media.

**Figure 13:**
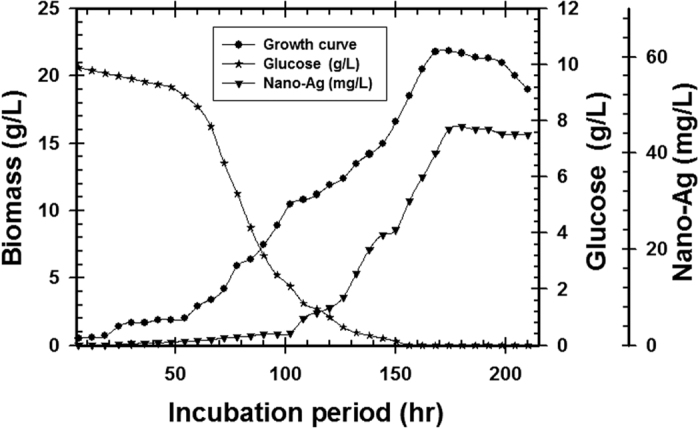
The relation between nano-Ag dry weight and the biomass growth profile of *T. harzianum* strain SYA.F4 which cultivated in medium (1).

As a general the success of industrial production for biological control agents depending not only the isolation, characterization & pathogenicity, but also on the successful mass production of the fungal cells in laboratory^66^,^69^,^70^. The selection of a carbon source able to enhance fungal growth without leading to catabolism is highly desirable, because a high concentration of microbial biomass is required in cultivations in order to maximize bioactive compounds & proteins productivity^71^. The relation between bioactive compound, protein secretion and the growth profile of the microorganism is therefore a key consideration for increased nanoparticles production. Hence, the growth medium could be one of the main factors affecting cells/protein production during a fermentation process^72^,^73^. Therefore, the use of high cell density cultivation is required in order to improve nano-Ag biosynthesis. To the best of our knowledge, there has been no work concerning the evaluation of such a strategy for improving of nano-Ag production which mycosynthesized by *T. harzianum*. Larger-scale cultivations were carried out under the selected steps; media evaluation, cultivation in 1.0 L shake-flask and cultivation in 7.0 L stirred tank bioreactor. So in the present study, firstly several available substrates of both organic, inorganic and mixed media were tested for mass multiplication of *T. harzianum* strain SYA.F4 to select the best medium to achieve high fungal growth. Among the tested media, medium (1) produced significantly higher 22 g/l of biomass production and 45.5 mg/l of nano-Ag dry mass weight was recorded (Figs 12 and 13).

#### Cultivation in 1L shake-flask

**Figure 14:**
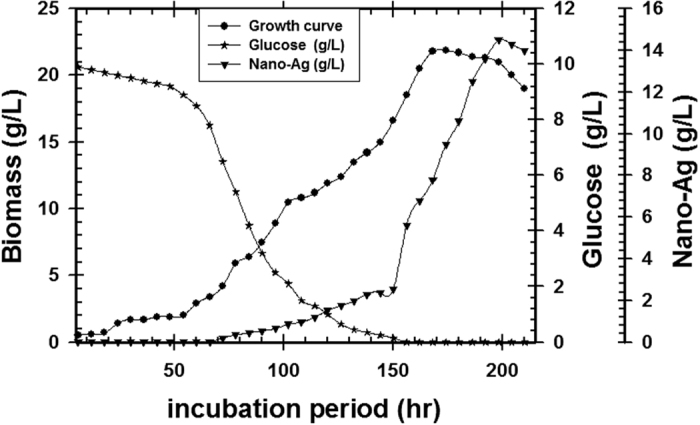
Time course of glucose consumption, nano-Ag production and biomass concentration on *T. harzianum* SYA.F4 cultivated in 1 L shake flask.

The resulted conditions for the pre-culture step in the selected medium were used to identify the time required for the complete consumption of glucose, attainment of the peak biomass concentration and nano-Ag production for inoculation of the 1 L shake flask. The *T. harzianum* SYA.F4 pre-culture was inculcated in 1 L shake flask (working volume 500 ml) to reach the initial biomass of 0.3 g/l. As shown in Fig. 14, an initial glucose concentration 10 g/l was used, and after 150 hr, the glucose was almost exhausted (0.15 g/l). By using kinetics measurements for fungal cells; we conclude that; μ_max_ was 0.045, Y_X_ (11.6), X _max_ was 23.2 g/l, T_d_ was 3.7 hr, P _max_ was 18.5 g/l; Y_P_ was 14 and incubation period was 210 hr.

#### High cell density cultivation in 7 L Stirred tank bioreactor

**Figure 15:**
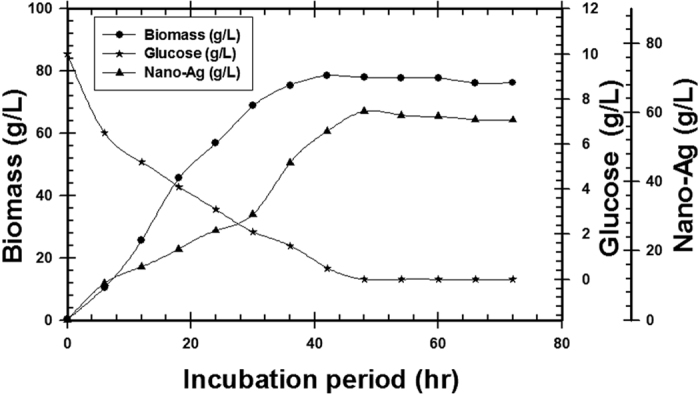
Time course of glucose consumption, nano-Ag production and biomass concentration on *T. harzianum* SYA.F4 cultivated in 7 L stirred tank bioreactor.

**Figure 16:**
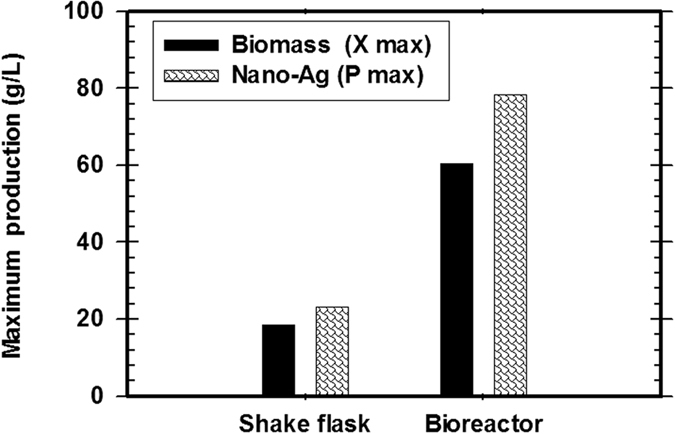
Comparison study between the basal reaction and optimized reaction indicated by final dry weight of nano-Ag (g/l) which myco-synthesized by *T. harzianum* strain SYA.F4 extract.

**Figure 17:**
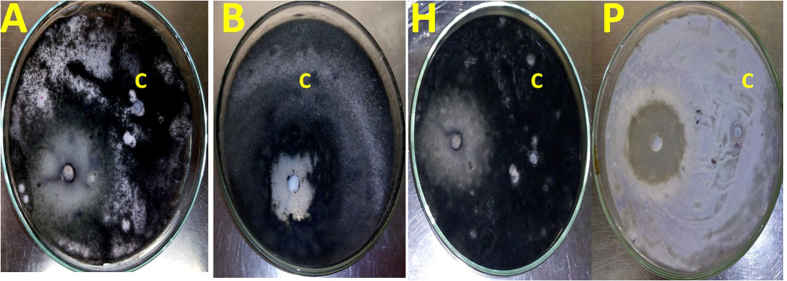
The Antifungal activity of nano-Ag (100 μg/ml) against some phytopathogenic fungi *in vitro*. (**A**) *Alternaria alternata*; (**B**) *Botrytis sp*.;(**H**) *Helminthosporium sp;* (**P**) *Phytophthora arenaria;* and (**C**); 0.01 M AgNO_3_ as a control.

**Figure 18:**
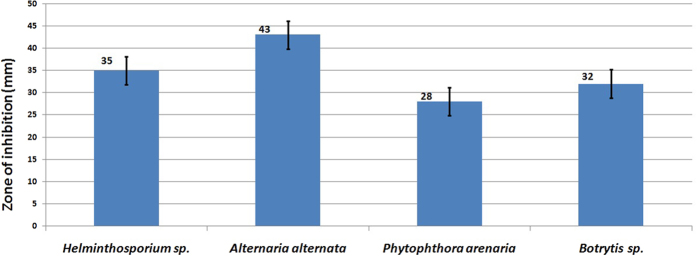
The chart shown the maximum ZOI (mm) produced from nano-Ag (100 μg/ml) against some phytopathogenic fungi.

The pre-culture of *T. harzianum* SYA.F4 were inculcated in 7 L stirred tank bioreactor (working volume 5 L) to reach the initial biomass of 0.3 g/l. In this experiment, by using kinetics measurements we conclude that; μ_max_ was 0.098, Y_X_ (30.5), X _max_ was 78.4 g/l, T_d_ was 0.3 hr, P _max_ was 60.5 g/l; Y_P_ was 42.6 and incubation period was 72 hr Fig. 15. Batch model prediction for biomass and nano-Ag production was higher than the shake flask experimental data at different stages of cell growth. The possible reason was that the fermentation conditions in stirred tank bioreactor (agitation, airflow and pH) were controlled Table 8 and Fig. 16. Antifungal activity of of nano-Ag by using different concentrations against some pytopathogenic fungi to determine the large inhibition zones was tested and calculated as shown in Figs 17 and 18 and Table 9. The concentration that formed large inhibition zones was 100 μg/ml which showed the highest antifungal activity against *Alternaria alternate* (43 mm) followed by *Helminthosporium sp.* (35 mm), *Botrytis sp*. (32 mm) and *Phytophthora arenaria* (28 mm).

## Discussion

Endophytes are the microorganisms that inhabit interior of plant tissues with symptomless disease to their host and are not host specific^42^,^74^. Different plants are gaining worldwide attention owing to the fact that the herbal bioactive compounds are cost effective, easily available and with negligible side effects. Endophytic fungi from plants are known as “Promising Source” of bioactive novel metabolites which has significant role in agriculture (management of plant diseases and promote plant growth) and in industries (have high production of specific enzymes or metabolite, high growth rate, easy handling in large-scale production and low-cost requirement for production procedures). These endophytic fungi are the rich source of functional metabolites such as alkaloids, amines, terpenoids, steroids, flavonoids, phenolic compounds; extracellular enzymes etc^75^ which used for biosynthesize metal nanoparticles have promising application in the field of agriculture, etc.^76^. Active metabolites from biological origin are produced by a large number of fungal species and most bioprospecting strategies were limited to some ecological groups of fungal species in Egypt^77^. Endophytic anamorphic Ascomycota have been used for green synthesis of AgNPs during the last decade worldwide^66^,^69^,^70^,^71^,^72^,^73^,^78^. Endophytic *Trichoderma* species may provide a range of benefits to their hosts. However, few studies have systematically examined the diversity of *Trichoderma* species associated with plant in tropical regions^79^. According to previous studies on *Trichoderma spp.,* the production of extracellular enzyme and nanoparticles in this fungus is more efficient than other fungi^66^,^69^. It is also shown that *Trichoderma spp.* has easier and cheaper cultivation requirements and higher growth rates on both industrial and laboratory scales, thereby having a lower cost in large-scale production^78^,^79^. It should be pointed out that large-scale production of silver nanoparticles by other techniques, such as chemical vapor deposition, irradiation, and liquid solution reduction, usually produces particles larger than a few micrometers in size^71^,^73^. There are very few studies carried on the biosynthesis of Ag NPs by endophytic *Trichoderma* strains (*T. reesei* and *T. viride*)^80^,^81^. In the present investigation, endophytic *Trichoderma* species were evaluated for the production of nano-Ag by reduction of AgNO_3_. All these isolates invariably produced nanoparticles which were evident from the change of color to dark brown. However the intensity of the color produced was highest for *Trichoderma* isolate F4. The most abundant *Trichoderma* isolate was used for further molecular identification using phylogenetic analysis which designated as *Trichoderma harzianum* strain SYA.F4 and deposited in NCBI GenBank (Accession Number KX084391).

Mycosynthesis of nano-Ag affected by the interaction of Ag NPs with biomolecules released by microorganism metabolism, likely proteins will influence surface chemistry of nanoparticles and modify their electronic charge and agglomeration state leading to the improvement of their biological activity^82^. Phytochemical analysis is carried out in plant species but only few reports are available in endophytes^47^. The ability of an endophyte to produce some metabolites but not others has been described by^16^ where different endophytes may produce different secondary metabolites hence play different functions in the mycosynthesis of nanoparticles. By using screening studies that has been done using different species of fungi for extracellular biosynthesis of Ag NPs, the reduction of silver ion to silver nanoparticles is associated with a NADH dependent reductase enzyme, and an electron shuttle (quinones or naphthoquinones) produced as secondary metabolite^83^,^84^. Peptides also appear to have a reductase-like activity due to their conformation. Also hydroxyls in the terpenoids oxidized to carbonyl groups and hence act as a reducing agent for silver ions. In this study, some the bioactive metabolites which produced from endophytic *T. harzianum* SYA.F4 and *T. harzianum* EMCC 540 strain determined and compared the secondary metabolites. From our results the highest dry mass weight of nano-Ag produced from extracellular fraction which extracted from *T. harzianum* SYA.F4 due to its have the highly nitrate reductase and protein concentration besides the presence of some phytochemical compound like Alkaloids, Flavonoids, Tannins, Phenols, and Cardiac glycosides; compared with *T. harzianum* EMCC 540 strain which have the highly carbohydrate concentration besides the presence of Alkaloids, Phenols and Terpenoids. These results supported by the previous reports that described the ability of an endophyte to produce some metabolites where different endophytes in a plant may produce different secondary metabolites hence play different functions in the plant and that the total number of metabolites in a plant extract may be a contribution of all the endophytes that live in the plant^16^,^47^. The synthesis of silver nanoparticles by different fungal species has been reported, but the exact mechanism of nanoparticle biosynthesis is still not well understood. The analysis and identification of active species in the nucleation and growth of metal nanoparticles is complex, mainly due to the interaction process along with microbial metabolic complexity. It is not clear why not all fungi are capable to synthesize active silver nanoparticles as antimicrobial agent^82^,^85^. From this studies; we reported that the antifungal activities of myco-synthesized nano-Ag from *T. harzianum* EMCC 540 observed; but the highly antifungal activities were detected by using nano-Ag produced from *T. harzianum SYA.F4*. The characteristics of the bactericidal and fungicidal effect of Ag NPs are dependent upon a number of properties, including particles size and size distribution, solubility and state of aggregation, elemental composition, mass and concentration, shape and crystal structure, surface area, charge, chemistry and the presence of impurities^85^,^86^. In the previous studies the superiority of the positively charged Ag NPs over the negatively charged particles, in terms of the antibacterial activity, was demonstrated^8^,^82^. The role of surface charge in their bacterial activity was assessed. Also the higher activity of Ag NPs is probably due to the proteins or other biocompatible materials adsorbed on the surface of these nanoparticles^67^,^87^. The adsorbed proteins and/or enzymes may augment the antimicrobial property of Ag NPs to some extent^9^,^82^,^88^.

From all the previous results we can conclude that; the endophytic *T. harzianum* SYA.F4 strain have mixed bioactive compounds and characters that made it very interested strain in industrial production of nano-Ag. The changes in extracellular fungal extract color from yellow to brown; indicated the formation of nano-Ag in the reaction mixture. This dark brown color is due to the surface plasmon resonance property of nano-Ag^2^. Many metals can be treated as free-electron system. These metals (plasma) contain equal numbers of positive ions and conduction free & highly mobile electrons. The free electrons are driven by the electric filed to oscillate coherently under the irradiation of an electromagnetic wave. These collective oscillations of the free electrons are called plasmons which can interact with visible light in phenomenon called surface plasmon resonance (SPR)^2^,^3^,^6^. SPR plays a major role in the determination of optical absorption spectra of metal nanoparticles, which shifts to a longer wavelength as the particle size increases^2^. The shape, size and several analytical analysis of the result nano-Ag were elucidated with the UV-Vis spectroscopy, EDX, XRD, FTIR, TEM and SEM. The UV-Vis spectroscopy results show strong surface plasmon resonance centered at 420 nm which indicates the formation of nano-Ag^3^,^6^. The separation between the spherical uniform shape nano-Ag (12.8 nm) seen in the TEM and SEM images could be due to capping of proteins and would explain the UV-Vis spectroscopy measurement, which is characteristic of well dispersed nanoparticles^3^. The elemental profile of mycosynthesized nano-Ag using *T. harzianum* strain SYA.F4 showed typical characteristic elemental peak at approximately 3 keV, which was attributed to the SPR of the metallic Ag nano-crystals and confirms the formation of Ag nanoparticles^6^. XRD pattern revealed six diffraction peaks could be indexed to (220), (111), (400), (200), (220), and (311) planes. As^3^ report; all the peaks corresponding to face-centered cubic (FCC) structure of metallic silver (JCPDS file no. 00–004–0783). Thus, XRD pattern obtained for the nano-Ag that revealed its crystalline nature and consisted with many earlier reports of the AgNPs synthesized by fungal extracts^43^.

Previously studies^43^,^47^ recorded that FTIR analysis from endophytic fungi revealed that the presence of polyphenols which act as bioreducing agents, while proteins could play a dual role as bioreducing and stabilizing agents^43^ reported that the carbonyl groups from the amino acid residues and proteins has the stronger ability to bind metal demonstrating that the proteins could possibly from the metal nanoparticles (capping of silver nanoparticles) to prevent agglomeration and thereby stabilize the medium. So the natural molecules could possibly perform dual functions of formation and stabilization of nano-Ag in the aqueous medium. Carbonyl groups proved that flavanones or terpenoids absorbed on the exterior of metal nanoparticles^89^. Flavanones or terpenoids could be adsorbed on the surface of metal nanoparticles, possibly by interaction through carbonyl groups in the absence of other strong ligating agents in sufficient concentration^47^,^89^ reported that, the nano-Ag solution, mycosynthesized by the bioreduction of Ag^+^ to Ag^−^ with *Trichoderma spp.,* is exceptionally stable. This stability is likely to be due to capping with proteins secreted by the fungus thus ensuring complete formation of stable nanoparticles. The nanoparticles were subjected to FTIR and phytochemical analysis for analyzing the capping ligand of nanoparticles which act as reducing and stabilizing agents. In this study FTIR analysis shown the intensive peaks which corresponding to amines, phenols, the secondary amide of the protein, methylene groups of the protein, aromatic-CH, C = O, -NHCO, C = C or the O–H bending mode, –CH3, aromatic amines, the ester group, C–O–C, and nano-Ag. The peak at 1025 cm^−1^ which assigned to C–OH of the phenols or carbohydrates which might has the stronger ability to reduce of Ag^+^ into Ag^0^. The precise reaction mechanism leading to the mycosynthesis of silver nanoparticles is not definitely realized yet^3^. Finally, we can conclude that the fungi which secrete much higher amount of bioactive substances may make it more suitable for the production of silver nanoparticles. In this regard, the results obtained in this work open several avenues of further studies.

Silver nanoparticles can be induced to have different forms depends on filtrate volume, salt concentration and physical conditions: pH, temperature and light intensity that affect the maximum yield, rate of synthesis and its size. There are many previous studies connected the stability and antibacterial activity of Ag NPs bio-fabricated from different endophytic fungi with pH and reported the optimum pH was 7^63^,^90^, pH 5^42^,^80^,^91^ and pH 3 reported by^92^,^93^,^94^,^95^,^96^. Finally^62^,^97^ recorded that biosynthesis of Ag NPs by using extracellular fraction of *Trichoderma reesei* and *T. harzianum* without adjusting pH and reported the formation of polydisperse spherical and occasionally ellipsoid Ag NPs in the size range from 5–63 nm at room temperature started within 3 hr. In this work by using some survey experiments on nano-Ag mycosynthesis reaction at different physical conditions (pH, temperature and light intensity), we observed the narrow ranges of pH and temperature (5–6 at 30–37 °C) in light or dark conditions (these results unpublished). So these physical conditions used constantly in all paper experiments.

They are many studies reported the optimized different physicochemical conditions by using one variables at time strategies to increase the nano-Ag particles rate and controlling its shape, size and charge^1^,^62^,^66^,^97^; while optimization of AgNPs mycosynthesis reaction by using Taguchi experimental designs and large scale production studies from *T. harzianum* were never reported up to our knowledge. Taguchi experimental designs are called “robust design” and it is a central theme of Taguchi Method. Therefore, Taguchi methodology is used to achieve a predictive knowledge of a complex, multi-variables process with the fewest possible trials and optimization of the experimental process itself^17^,^21^,^22^. In this work; Taguchi structured approach was used to determining the best combination of inputs to produce a dry weight of nano-Ag based on a design of experiments (DOE) methodology for determining parameter levels such as the precursor concentration, reductant concentration and agitation speed. Taguchi method is applied to analyze the mean response for each run in the inner array and to analyze the variation using as appropriately chosen S/N ratio which are different according to the type of the characteristics. Finally, the physicochemical parameters for nano-Ag production are the precursor conc., at 0.01 M, the diluted reductant10 (v/v) adjusted pH to 5 and incubated at 30 °C in 200 rpm for 24 hr. we can conclude the nano-Ag production increased (3 times larger than basal condition) and improved through the Taguchi method approach.

For industrial applications, fungi should have certain properties which include high production of specific metabolite, high growth rate; easy handling in large-scale production and low-cost requirement for production procedures^33^. The production and quality of bioactive compounds from endophytic fungi depends on natural conditions of the association and the nature of the organic or synthetic medium used. Strategies can be developed to use these fungi for exploitation of bioactive compounds^10^. Fungi secrete large amounts of enzymes and are easy growing on every medium so they are considered as a proper choice for the biosynthesis of nanoparticles^27^. Many studies have been done so far using various species of fungi for the biosynthesis of Ag NPs such as *Aspergillus, Fusarium, Penicillium, Trichoderma*, and *Cladosporium*^1^,^66^. Many reports have appeared on enzymes production by the submerged cultivation of *T. reesei* using pretreated lignocellulosic biomass as the carbon source like baggase, vegetable waste, and wheat grains etc.^66^,^67^. Among minerals, fungi require nitrogen in the largest amounts, so nitrogen can be considered as the limiting factor for their growth can be accounted. Unlike bacteria, fungi cannot fix atmospheric nitrogen, but they are able to use many other forms of nitrogen like amino acids, ammonium, and nitrate^7^. The precise reaction mechanism leading to the biosynthesis of sliver nanoparticles is yet to be clarified^1^,^7^,^66^. Present investigation was carried out to evaluate available cheaper medium for mass multiplication of *T. harzianum* strain SYA.F4 for mycosynthesis of nano-Ag as anti-phytopathogenic agent by using submerged batch fermentation mode. Previously several available of organic and inorganic or mixed media were tested for fungal mass production; in this study the best medium which achieves high fungal mass weight was selected and the relationship between the fungal biomass production and mycosynthesized nano-Ag also studied. By using semi inorganic medium biomass production (60.5 g/l) and nano-Ag dry weight (78.4 g/l) were recorded by using large-scale batch cultivation in 7 L stirred tank bioreactor; so we can conclude that nano-Ag can be induced to get to the maximum mass weight depends on the fungal culture medium and quantity of its biomass. The possible reason may be related to the fermentation conditions in stirred tank bioreactor (agitation, airflow and pH) were controlled^27^,^34^.

The results of the nano-Ag as antifungal agent were consisted with few earlier reports^29^,^31^,^89^. The important advantages of Ag NPs-based antimicrobial agents are their biocompatibility, health and environmental safety, and their excellent stability^63^,^89^ reported that; the mycosynthesized AgN Ps at (5 and 10 ppm) exhibited a more effective activity in inhibiting the mycelia growth of pathogenic strains of *Alternaria solani*, the causal agent of tomato early blight disease as compared to the recommended chemical fungicide used (Ridomil gold plus 2 g/l). Damage of fungal hyphae structure was observed through electron microscopy in hyphae treated with the mycosynthesized Ag NPs^7^,^63^. A simple, fast, cost-effective, eco-friendly and stable method for mycogenic synthesis of nano-Ag was successfully developed in the present work by using endophytic *T. harzianum* SYA.F4 which isolated from healthy organic tomato plant. As mentioned in our results; 100 μg/ml of nano-Ag showed the highest inhibition zones against *A.alternate* followed by *Helminthosporium sp., Botrytis sp*. and *P. arenaria*. The mechanism of nano-Ag antifungal activity may be related to damaging the fungus membrane lipid bilayer, leading to intracellular ion efflux resulting in cell death. Also, accumulation of Ag NPs in the cell nuclei and interaction with DNA may lead to cell death^29^,^63^. Fairly the biochemical mechanisms for the studies of the production of Ag NPs especially from endophytic fungi have some aspects were left open, which are likely to be taken up into future research to further clarify, such as the understanding of the compounds that are involved on the Ag NPs and its fungicidal effect.

## Additional Information

**How to cite this article:** EL-Moslamy, S. H. *et al*. Applying Taguchi design and large-scale strategy for mycosynthesis of nano-silver from endophytic *Trichoderma harzianum* SYA.F4 and its application against phytopathogens. *Sci. Rep.* **7**, 45297; doi: 10.1038/srep45297 (2017).

**Publisher's note:** Springer Nature remains neutral with regard to jurisdictional claims in published maps and institutional affiliations.

## Figures and Tables

**Table 1:** Phytochemical investigations of active constituents in endophytic *Trichoderma harzianum* strain SYA.F4 and *T. harzianum* EMCC 540 extract (ethyl acetate solvent), +Presence; −absence, repeated the experiments three times for each replicates.

	*T. harzianum* SYA.F4	*T. harzianum* EMCC 540
Alkaloids	++	++
Flavonoids	+	−
Tannins	+	−
Phenols	+++	+
Steroids	−	−
Saponins	−	−
Terpenoids	−	++
Cardiac glycosides	+	−

**Table 2:** The quantitative measurements of secondary metabolites produced from endophytic *T. harzianum SYA.F4* compared with others from *T. harzianum* EMCC 540.

Secondary metabolites	*T. harzianum* SYA.F4	*T. harzianum* EMCC 540
Carbohydrate concentration (μg/μl)	25	75
Protein concentration (g/l)	2.5	1.2
Nitrate reductase (nmol/h/ml)	320	200

**Table 3:** Factors and their levels employed in Taguchi’s experimental designs matrix (L16 (4^3^)) for optimization the nano-Ag mycosynthesis reaction parameters.

Factors	levels			
	1	2	3	4
Precursor conc (M)	0.01	0.5	1.0	1.5
Reductant conc (v/v)	10%	50%	80%	100%
Agitation speed (RPM)	50	100	150	200

**Table 4:** Taguchi’s L16 (4^3^) Orthogonal array design experimental setup for optimization the nano-Ag mycosynthesis reaction parameters, the final dry weight of nano-Ag and the average S/N ratio of each factor at each level.

Trail	Precursor conc. (M)	Reductant conc. (%)	Agitation speed (RPM)	Nano-Ag (mg/L)	*Predicted Nano-Ag (mg/L)*	S/N ratio(dB)
L1	1	1	1	28	28.018	28.94
L2	1	2	2	26	26.503	28.30
L3	1	3	3	25	24.988	27.96
L4	1	4	4	23.59	23.473	27.45
L5	2	1	2	28	28.4735	28.94
L6	2	2	1	24.63	24.8985	27.83
L7	2	3	4	25.215	25.4435	28.03
L8	2	4	3	22	21.8685	26.85
L9	3	1	3	30	28.929	29.54
L10	3	2	4	28	27.414	28.94
L11	3	3	1	23.24	21.779	27.32
L12	3	4	2	20	20.264	26.02
L13	4	1	4	28.465	29.3845	29.09
L14	4	2	3	26	25.8095	28.30
L15	4	3	2	22	22.2345	26.85
L16	4	4	1	18	18.6595	25.11

**Table 5:** Regression Statistics resulted from linear multiple regression analysis method for variables affecting nano-Ag myco-synthesis parameters.

Regression Statistics	
Multiple R	0.98
R Square	0.96
Adjusted R Square	0.95
Standard Error	0.68
Observations	16

**Table 6:** Statistical analysis of Taguchi design showing coefficient, *t-*test values, *P-*values and confidence level (%) for variables affecting nano-Ag myco-synthesis parameters from *T. harzianum* strain SYA.F4.

Variables	Coefficients	Standard Error	t Stat	P-value	Confidence level (%)
Precursor conc. (M)	−0.5745	0.154101157	−3.72807064	0.002884064	99.7
Reductant conc. (v/v)	−2.545	0.154101157	−16.51512581	1.28711E-09	99.9
Agitation speed (RPM)	1.03	0.154101157	6.683921253	2.24754E-05	99.9

**Table 7:** ANOVA test analysis for Taguchi design experiments.

	df	SS	MS	Significance F
Regression	3	157.359505	52.45316833	5.3E-09
Residual	12	5.69932	0.474943333	
Total	15	163.058825		

**Table 8:** Comparison study between the batch cultivations in shake flask and in stirred tank bioreactor indicated by *T. harzianum* strain SYA.F4 growth conditions and biomass & nano-Ag production kinetics.

Growth conditions and biomass & nano-Ag production kinetics	Batch in shake flask	Batch in stirred tank bioreactor
Container volume (ml)	1000	7000
Working Volume (ml)	500	5000
pH	Un-controlled	5.5
Aeration (Bar)	Un-controlled	Adjusted to let the dissolved O_2_ level did not below 30%
Agitation (RPM)	200	Adjusted to let the dissolved O_2_ level did not below 30%
Incubation period (hr)	210	72
Specific growth rate (μ_max_)	0.049 (l/hr)	0.098 (l/hr)
Biomass yield coefficient (Y_X_)	11.6 (g/g)	30.5 (g/g)
Maximum biomass (X _max_)	23.2 g/l	78.4 g/l
Doubling time (t_d_)	3.7 hr	0.3 hr
Maximum Product (P _max_)	18.5 g/l	60.5 g/l
Product yield coefficient (Y_P_)	14 (g/g)	42.6 (g/g)

**Table 9:** Detection of the maximum inhibition zone produced from application of nano-Ag as antifungal agent against some phytopathogenic fungi *in vitro.*

Phytopathogenic fungi	Pure mycosynthesized nano-Ag			
	Zone of inhibition (mm)			
	50 μg/ml	100 μg/ml	150 μg/ml	200 μg/ml
*Helminthosporium sp.*	15	35	32	20
*Alternaria alternata*	24	43	42	10
*Phytophthora arenaria*	13	28	25	20
*Botrytis sp.*	25	32	30	27
